# Effectiveness of Multisensory Exercises Versus Conventional Balance Exercises on Balance and Coordination in the Geriatric Population: A Systematic Review

**DOI:** 10.7759/cureus.113574

**Published:** 2026-07-29

**Authors:** Anisha D Gulati, Suraj Kanase

**Affiliations:** 1 Department of Physiotherapy, Krishna College of Physiotherapy, Krishna Vishwa Vidyapeeth (Deemed to be University), Karad, IND

**Keywords:** and physiotherapy, balance training, coordination, falls prevention, geriatrics, multisensory rehabilitation, postural control, reaction time

## Abstract

Balance impairment is a major contributor to falls, morbidity, and loss of independence in older adults. Although conventional balance training is widely used, recent attention has shifted toward multisensory interventions, including balance tasks, dual-task training, and perturbation-based exercises, which target the visual, vestibular, and proprioceptive systems and may yield superior outcomes by improving sensory integration and postural control. In light of the potential for improved outcomes with multisensory interventions, this review systematically evaluates and compares the effectiveness of multisensory balance training, which integrates visual, auditory, and somatosensory cues, with conventional balance exercises, which typically involve standard physical activities without additional sensory input, in improving balance, coordination, and reaction time among individuals aged 60 years and older. To address this objective, this systematic review was conducted in accordance with the Preferred Reporting Items for Systematic Reviews and Meta-Analyses (PRISMA 2020) guidelines. Comprehensive searches were performed in PubMed, Scopus, CINAHL, EBSCO, and Cochrane CENTRAL for studies published within the past 10 years. Eligible studies included randomized controlled trials involving geriatric participants. Data extraction and qualitative synthesis were undertaken, and the risk of bias was assessed using the Cochrane Risk of Bias 2.0 tool. As a result of these search and selection methods, 12 studies with a total of 958 participants were included. The multisensory interventions evaluated included proprioceptive training (e.g., joint-position exercises), perturbation-based training (e.g., unexpected platform movements), visual-cue training (e.g., visual markers for movement), posturography-based training (e.g., feedback from force platforms), and unstable-surface training (e.g., balance boards). These interventions demonstrated greater improvements in balance and functional mobility compared to conventional exercises in most studies. Significant improvements were observed in Berg Balance Scale (BBS), Timed Up and Go (TUG), and Functional Reach Test (FRT) outcomes (p < 0.05 to p < 0.001). Risk of bias assessment indicated that nine studies had some concerns, while three were classified as high risk. Based on these findings, multisensory balance training appears more effective than conventional exercise in improving balance, coordination, and functional mobility in the geriatric population. Integration of these approaches into physiotherapy practice is recommended; however, further high-quality studies are needed to confirm these findings.

## Introduction and background

Balance impairment is a significant health concern among older adults and is closely linked to increased risks of falls, injury, hospitalization, and reduced quality of life. Falls are a leading cause of morbidity and mortality in the geriatric population. They often result in fractures, disability, and loss of independence. About one-third of individuals aged 65 years and older experience at least one fall each year. This underscores the clinical importance of balance dysfunction in this demographic [1].

Aging leads to progressive degeneration of several physiological systems needed for postural control, including the visual, vestibular, and proprioceptive systems. The visual system provides environmental orientation, the vestibular system detects head movement and spatial orientation, and the proprioceptive system provides feedback on joint position and movement. Age-related decline in these systems results in impaired sensory integration, delayed motor responses, and decreased postural stability, thereby increasing the likelihood of falls and functional limitations [2,3].

Conventional balance training is widely used in physiotherapy and typically emphasizes strengthening exercises, static postural control, and basic dynamic activities. These interventions aim to enhance muscle strength, joint stability, and coordination. However, conventional approaches may not fully address the complex sensory integration required for real-world balance, in which multiple sensory inputs must be processed simultaneously [4].

Recently, multisensory balance training has emerged as a comprehensive rehabilitation approach. These interventions are designed to simultaneously stimulate and integrate visual, vestibular, and proprioceptive inputs, thereby enhancing neuromuscular coordination and adaptive postural responses. Techniques such as proprioceptive training on unstable surfaces, visual-cue-based exercises, and perturbation-based training challenge the sensory systems and promote adaptability in the central nervous system. Multisensory exercise has emerged as a promising intervention for improving balance and functional performance in older adults. However, the available evidence comparing its effectiveness with conventional exercise interventions remains variable, highlighting the need for a systematic evaluation [5,6].

Evidence supporting multisensory interventions is increasing, but findings across studies remain heterogeneous because of variations in study design, intervention protocols, outcome measures, and sample characteristics. As a result, while some studies report significant superiority of multisensory training, others find outcomes comparable to conventional exercises. This variability makes it difficult to draw definitive conclusions and highlights the need for a systematic evaluation of the available evidence [7-9].

This systematic review aimed to determine whether multisensory balance training is more effective than conventional balance exercises in improving balance, coordination, and reaction time among older adults.

## Review

Methodology

Study Design

This systematic review was conducted in accordance with the Preferred Reporting Items for Systematic Reviews and Meta-Analyses (PRISMA 2020) guidelines [10], which provide a standardized framework for reporting systematic reviews and ensure methodological transparency and reproducibility. The review was registered in the International Prospective Register of Systematic Reviews (PROSPERO), with the registration number CRD420251127845, before commencement. The review followed a structured, multi-step process that included the following components:

Protocol and Planning

A predefined methodological approach was developed prior to conducting the review. Formal protocol registration (e.g., PROSPERO) was completed, and the review strictly adhered to PRISMA recommendations to minimize bias and enhance transparency. 

Search Strategy

A comprehensive literature search was conducted in PubMed, Scopus, CINAHL, and Cochrane CENTRAL for studies published within the past 10 years. The database search was conducted between October 2025 and December 2025. Search terms included combinations of "multisensory training," "balance training," "proprioceptive training," "elderly," "geriatric," and "postural control." Boolean operators (AND/OR) were used to refine the search. Only English-language, human studies were included.

PubMed (2,547) search string: (((("Treatment Outcome"[Mesh]) OR "Comparative Effectiveness Research"[Mesh]) OR "Treatment Outcome"[Text Word] OR "Comparative Effectiveness Research"[Text Word] OR effectiveness[Text Word] OR efficacy[Text Word] OR effect[Text Word] OR impact[Text Word] OR effective[Text Word] OR outcome[Text Word] OR intervention[Text Word] OR performance[Text Word]) AND ((((Proprioceptive training[MeSH Terms])) OR (Balance Training[MeSH Terms])) OR ("Proprioceptive training"[Text Word] OR "Balance Training"[Text Word] OR "Sensory Training"[Text Word] OR "Multisensory Exercise*"[Text Word] OR "Multisensory Training"[Text Word] OR "Sensorimotor Training"[Text Word] OR "Balance Training"[Text Word] OR "Vestibular Stimulation"[Text Word] OR "Somatosensory Training"[Text Word] OR "Visual Motor Integration"[Text Word] OR "Postural Control Training"[Text Word] OR "Coordination Exercise*"[Text Word] OR "Motor Control Training"[Text Word]))) AND ((aged[MeSH Terms]) OR ("Aged"[Title/Abstract] OR "Older Adult*"[Title/Abstract] OR "Elder*"[Title/Abstract] OR Geriatric[Title/Abstract] OR Aging[Title/Abstract] OR "Senior Citizen*"[Title/Abstract] OR "Older People"[Title/Abstract] OR "Elderly Individual*"[Title/Abstract] OR "Older Person*"[Title/Abstract] OR "Geriatric Patient*"[Title/Abstract] OR "Elder Adult*"[Title/Abstract]))

Scopus (2,682) search string: (TITLE-ABS-KEY("Treatment Outcome" OR "comparative effectiveness research" OR effectiveness OR efficacy OR effect OR impact OR effective OR outcome OR intervention OR performance)) AND (TITLE-ABS-KEY("Proprioceptive training" OR "Balance Training" OR "Sensory Training" OR "Multisensory Exercise*" OR "Multisensory Training" OR "Sensorimotor Training" OR "Proprioceptive Training" OR "Balance Training" OR "Vestibular Stimulation" OR "Somatosensory Training" OR "Visual Motor Integration" OR "Postural Control Training" OR "Coordination Exercise*" OR "Motor Control Training")) AND (TITLE-ABS-KEY("Aged" OR "Older Adult*" OR "Elder*" OR Geriatric OR Aging OR "Senior Citizen*" OR "Older People" OR "Elderly Individual*" OR "Older Person*" OR "Geriatric Patient*" OR "Elder Adult*"))

Cochrane (1,177) search string: ("Proprioceptive training"):ti,ab,kw("Balance Training"):ti,ab,kw ("Sensory Training"):ti,ab,kw ("Multisensory Exercise"):ti,ab,kw ("Multisensory Training"):ti,ab,kw("Sensorimotor Training"):ti,ab,kw ("Vestibular Stimulation"):ti,ab,kw ("Somatosensory Training"):ti,ab,kw ("visual motor integration"):ti,ab,kw ("Postural Control Training"):ti,ab,kw ("Coordination Exercise"):ti,ab,kw ("Motor Control Training"):ti,ab,kw {OR #1-#12} MeSH descriptor: [Treatment Outcome] explode all trees (outcome):ti,ab,kw (impact):ti,ab,kw (efficacy):ti,ab,kw (effect*):ti,ab,kw (performance):ti,ab,kw ("comparative effectiveness research"):ti,ab,kw {OR #14-#20} MeSH descriptor: [Aged] explode all tree ("older adults"):ti,ab,kw (elder*):ti,ab,kw (aging):ti,ab,kw (geriatric):ti,ab,kw ("senior citizen"):ti,ab,kw ("Older People"):ti,ab,kw ("Older Person"):ti,ab,kw {OR #22-#29}#13 AND #21 AND #30 

CINAHL (125) search string: ((MH "Treatment Outcomes+" OR MH "Comparative Effectiveness Research" OR (TI(effectiveness OR efficacy OR effect OR impact OR "treatment outcome*" OR intervention*) OR AB(effectiveness OR efficacy OR effect OR impact OR "treatment outcome*" OR intervention*))) 

AND 

(MH "Proprioceptive Training" OR MH "Balance Training" OR TI("Sensory Training" OR "Multisensory Exercise*" OR "Multisensory Training" OR "Sensorimotor Training" OR "Proprioceptive Training" OR "Balance Training" OR "Vestibular Stimulation" OR "Somatosensory Training" OR "Visual-Motor Integration" OR "Postural Control Training" OR "Coordination Exercise*" OR "Motor Control Training") 

OR AB("Sensory Training" OR "Multisensory Exercise*" OR "Multisensory Training" OR "Sensorimotor Training" OR "Proprioceptive Training" OR "Balance Training" OR "Vestibular Stimulation" OR "Somatosensory Training" OR "Visual-Motor Integration" OR "Postural Control Training" OR "Coordination Exercise*" OR "Motor Control Training")) 

AND 

(MH "Exercise Therapy+" OR MH "Physical Therapy Modalities+" OR MH "Postural Balance" OR TI("Conventional Exercise*" OR "Traditional Exercise*" OR "Standard Balance Training" OR "Static Balance Training" OR "Dynamic Balance Exercise*" OR "Physical Rehabilitation" OR "Therapeutic Exercise" OR "Physiotherapy Exercise*" OR "Balance Re-education") 

OR AB("Conventional Exercise*" OR "Traditional Exercise*" OR "Standard Balance Training" OR "Static Balance Training" OR "Dynamic Balance Exercise*" OR "Physical Rehabilitation" OR "Therapeutic Exercise" OR "Physiotherapy Exercise*" OR "Balance Re-education")) 

AND 

(MH "Aged+" OR TI("Older Adult*" OR Elderly OR "Geriatric Population" OR Aging OR "Senior Citizen*" OR "Older People" OR "Elderly Individual*" OR "Older Person*" OR "Geriatric Patient*" OR "Elder Adult*") 

OR AB("Older Adult*" OR Elderly OR "Geriatric Population" OR Aging OR "Senior Citizen*" OR "Older People" OR "Elderly Individual*" OR "Older Person*" OR "Geriatric Patient*" OR "Elder Adult*")))

Study Selection Process

Selection was performed independently by two primary reviewers and a senior research/academic faculty member. Disagreements that could not be resolved by consensus were referred to a third researcher for final adjudication. The selection of studies was carried out in three stages: Title screening - Irrelevant studies were excluded based on the title; Abstract screening - Studies that did not meet the inclusion criteria were removed; and Full-text review - Eligible studies were assessed in detail.

**Figure 1:**
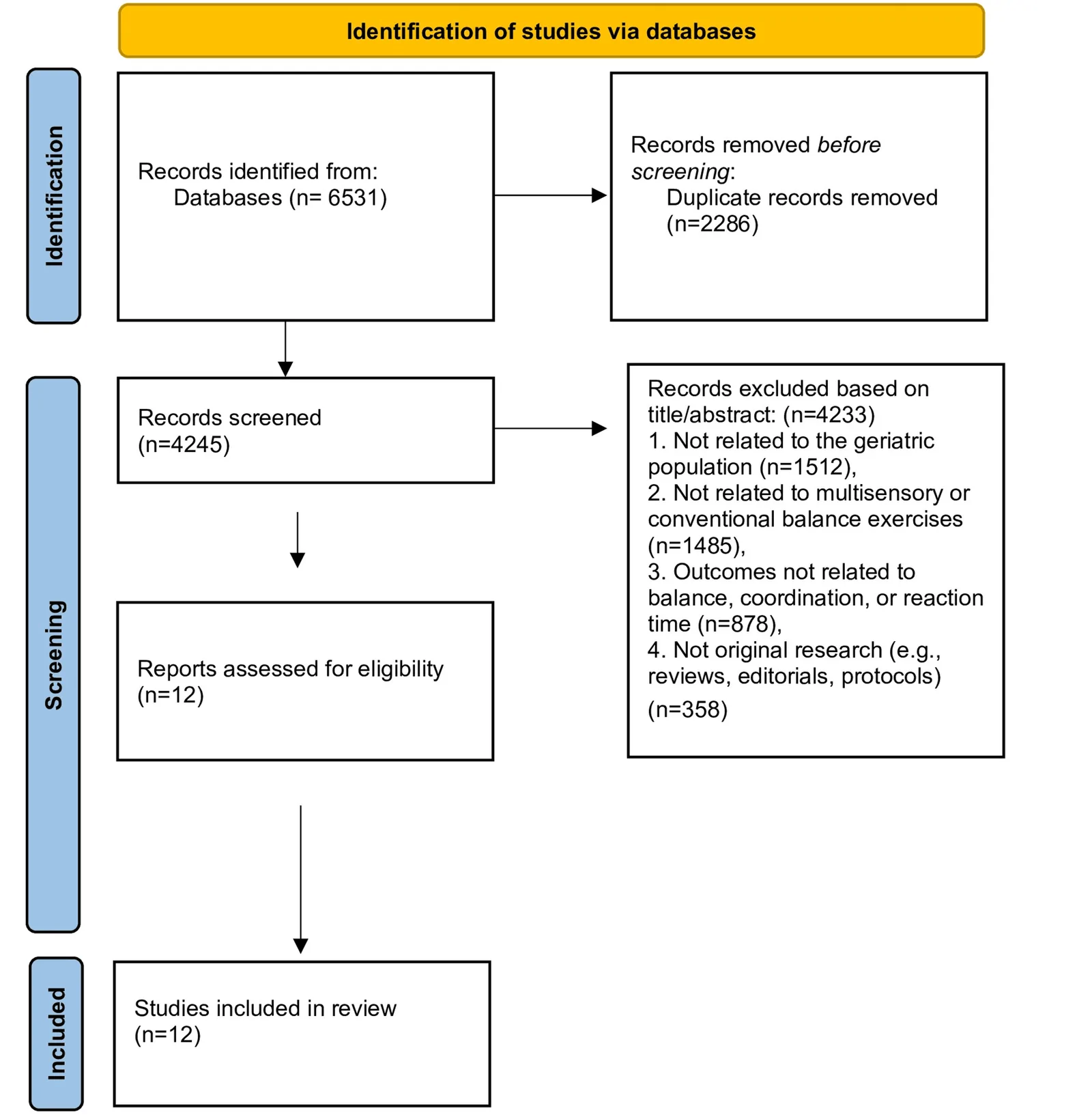
PRISMA flowchart PRISMA: Preferred Reporting Items for Systematic Reviews and Meta-Analyses [10]

The study selection process was documented in a PRISMA flow diagram, which illustrated the number of records identified, screened, excluded, and included in the final review (Figure 1).

Inclusion Criteria

The following studies are included the following: (1) studies including community-dwelling or institutionalized older adults aged 60 years and above; studies evaluating multisensory balance training interventions (e.g., proprioceptive, vestibular, visual cue, perturbation-based, or unstable surface training) compared with conventional balance exercises or standard physiotherapy programs; randomized controlled trials (RCTs) published in peer-reviewed journals; and studies reporting at least one standardized balance, coordination, mobility, or reaction time outcome measure, including the Berg Balance Scale (BBS), Timed Up and Go Test (TUG), Functional Reach Test (FRT), Dynamic Gait Index (DGI), or Tinetti Performance-Oriented Mobility Assessment. It also included studies published in the English language within the last 10 years: human studies involving geriatric participants with balance impairments or increased fall risk. For this review, multisensory exercise was defined as any structured exercise intervention that simultaneously challenged or integrated two or more sensory systems involved in postural control, including visual, vestibular, and somatosensory/proprioceptive inputs. Eligible interventions included balance training performed on unstable surfaces, sensory integration exercises, perturbation-based training, dual-task balance exercises involving sensory challenges, or comparable programs designed to enhance multisensory integration. Interventions targeting only a single sensory system or conventional balance exercises without a multisensory component were not classified as multisensory training.

Exclusion Criteria

The following studies are excluded: studies involving neurological conditions not primarily related to balance; non-interventional studies (e.g., observational studies and reviews); case reports; and conference abstracts. Studies involving participants with neurological disorders that independently influence balance and mobility, including stroke, Parkinson's disease, multiple sclerosis, spinal cord injury, vestibular disorders, and other central neurological conditions, were excluded to minimize clinical heterogeneity and to ensure that the observed effects were attributable to multisensory exercise interventions in older adults with age-related balance impairment.

Data Synthesis

The findings of this systematic review suggest that multisensory exercise may provide additional benefits for improving balance and functional mobility in older adults compared with conventional exercise. However, given the heterogeneity of the included studies, these findings should be interpreted with caution. Further high-quality, adequately powered RCTs with standardized intervention protocols and outcome measures are needed to confirm these observations.

Result

A total of 12 studies involving 958 participants were included in this systematic review, with sample sizes ranging from 17 to 281 participants. Among the included studies, 10 reported significantly greater improvements in the multisensory intervention groups than in the conventional balance exercise groups, while two demonstrated improvements in both groups with comparatively smaller between-group differences (see Table 1). Multisensory interventions - including proprioceptive training, perturbation-based exercises, visual cue integration, posturography training, and unstable surface training - consistently demonstrated superior improvements in dynamic balance, functional mobility, and reactive postural control (Table 1). Significant reductions in TUG scores and improvements in BBS and FRT scores were reported across most studies, with p-values ranging from <0.05 to <0.001 (see Table 1). Foam surface and perturbation-based training demonstrated the greatest improvements in postural stability and coordination (see Table 1). The BBS, TUG, FRT, DGI, and Tinetti Performance-Oriented Mobility Assessment were the most frequently used outcome measures. Risk of bias assessment using the Cochrane Risk of Bias 2.0 tool [11] showed that nine studies had "some concerns," primarily due to lack of blinding and unclear allocation concealment, while three studies were classified as high risk of bias due to methodological limitations, such as non-randomized designs and inadequate control procedures (see Table 2).

**Table 1:** Summary of the 12 included articles BBS, Berg Balance Scale; DGI, Dynamic Gait Index; FRT, Functional Reach Test; MFS, Morse Fall Scale; OLS, One-Leg Stance; OLST, One-Leg Stance Test; TST, Tandem Stance Test; TUG, Timed Up and Go Test

Author & Year	Sample Size	Intervention	Outcome Measures	Results
Hirase et al., 2015 [12]	n = 93	Balance training on foam (unstable) vs. a stable surface vs. control	OLST, TST, TUG, Chair Stand	Significant improvement in all outcomes (p < 0.001); the foam group showed earlier & greater improvement vs. others.
					Jarret et al., 2015 [13]	n = 174	Standard rehab + Otago exercises vs usual care	Balance tests, fall-related outcomes	Greater balance improvement in intervention group; short-term benefits observed
										Sabat et al., 2022 [14]	n = 81	Balance & coordination on different platforms	BBS, FRT, and coordination tests	All improved; the foam & Swiss ball were superior; the foam showed the greatest improvement.
Sunny et al., 2017 [15]	n = 40	Balance training + visual cues vs balance training alone	BBS, DGI	Significant improvement in both; the visual cue group was superior (p<0.001).										
Brach et al., 2017 [16]	n = 281	Group-based timing & coordination exercises vs usual activity	BBS, TUG	Significant improvement in balance & mobility in the intervention group (p < 0.05)										
					Lacroix et al., 2015 [7]	n = 66	Supervised vs. home-based balance and strength training	Balance tests, muscle power	Both improved; the supervised group showed greater gains (p<0.05).
										Chien et al., 2018 [5]	n = 17	Perturbation-based balance training	Postural sway, balance control	Significant improvement in reactive & dynamic balance (p < 0.05)
Siddiqui et al., 2017 [17]	n = 40	Posturography training vs. conventional balance training	BBS, TUG	Both improved; the posturography group was superior (p < 0.05).										
					Balakrishnan et al., 2023 [18]	n = 50	Structured balance training program	Balance scales, equilibrium measures	Significant improvement in balance and equilibrium (p < 0.05)					
										Nematollah et al., 2016 [19]	n = 44	Task-oriented vs. sensory vs. combined training	Balance performance scales	All improved; combined training was most effective.
Espejo-Antúnez et al., 2020 [20]	n = 42	Proprioceptive exercises vs. conventional therapy	TUG, Tinetti, OLS, MFS	Significant improvement in balance & function vs. control										
					Vaishnavi et al., 2021 [21]	n = 30	Balance training vs. conventional exercise	BBS, TUG	Both improved; balance training is superior.					

**Table 2:** Risk of bias assessment using the Cochrane Risk of Bias 2.0 tool

Study No.	Study Title	Domain 1: Randomization process	Domain 2: Deviations from intended interventions	Domain 3: Missing outcome data	Domain 4: Measurement of the outcome	Domain 5: Selection of the reported result	Overall RoB
1	Improving Balance in Older People: A Double-Blind Randomized Clinical Trial of Three Modes of Balance Training [19]	Low	Low	Low	Low	Some concerns	Some concerns
2	The Effect of Proprioceptive Exercises on Balance and Physical Function in Institutionalized Older Adults: A Randomized Controlled Trial [20]	Low	Some concerns	Low	Some concerns	Low	Some concerns
3	To Compare the Effectiveness of Balance Training and Conventional Exercises for Elderly Individuals [21]	Some concerns	Some concerns	Low	Some concerns	Some concerns	Some concerns
4	Effects of a Balance Training Program Using a Foam Rubber Pad in Community-Based Older Adults: A Randomized Controlled Trial [12]	Some concerns	Some concerns	Low	Some concerns	Some concerns	Some concerns
5	Can a Three-Week Program in a Rehabilitation Center Improve Balance in Elderly People? A Randomized Clinical Controlled Trial [13]	Some concerns	Some concerns	Low	Some concerns	Some concerns	Some concerns
6	Comparing the Effect of Balance and Coordination Exercise on Different Platforms as on Floor, Swiss Ball & Foam in Geriatric Population – A Randomized Control Trial [14]	Some concerns	Some concerns	Low	Some concerns	Some concerns	Some concerns
7	Effect of Visual Cue Training on Balance and Walking in Elderly: A Single-Blind Randomized Controlled Trial [15]	Some concerns	Some concerns	Low	Low	Some concerns	Some concerns
8	Effectiveness of a Timing and Coordination Group Exercise Program to Improve Mobility in Community-Dwelling Older Adults: A Randomized Clinical Trial [16]	Some concerns	Some concerns	Low	Some concerns	Some concerns	Some concerns
9	Effects of a Supervised versus an Unsupervised Combined Balance and Strength Training Program on Balance and Muscle Power in Healthy Older Adults: A Randomized Controlled Trial [7]	Some concerns	Some concerns	Low	Some concerns	Some concerns	Some concerns
10	Effects of Dynamic Perturbation-Based Training on Balance Control of Community-Dwelling Older Adults [5]	High	Some concerns	Low	Some concerns	Some concerns	High
11	Effects of Dynamic Posturographic Balance Training Versus Conventional Balance Training on Mobility and Balance in the Elderly [17]	Some concerns	Some concerns	Low	Some concerns	Some concerns	Some concerns
12	Benefits of Balance Training to Maintain Equilibrium Among Old-Age People [18]	High	Some concerns	Low	Some concerns	Some concerns	High

Study Characteristics

A total of 12 studies involving 958 participants were included in this review. The sample sizes ranged from 17 to 281 participants, reflecting variability in study scale and design. The included studies comprised RCTs (see Table 1). The interventions evaluated across studies included multisensory training approaches, such as proprioceptive training, perturbation-based exercises, visual cue integration, posturography, and training on unstable surfaces (e.g., foam surfaces and Swiss balls). Conventional balance training included static and dynamic balance exercises, strengthening exercises, and standard physiotherapy protocols. The duration and frequency of interventions varied across studies, contributing to methodological heterogeneity.

Outcome Measures

The most commonly used outcome measures across studies were the following: BBS, which assesses static and dynamic balance; TUG, which evaluates functional mobility and fall risk; and FRT, which measures dynamic balance. Additional measures included postural sway analysis, the DGI, and the Tinetti Performance-Oriented Mobility Assessment. The use of these standardized tools allowed for partial comparability of results across studies (see Table 1).

Synthesis of Findings

All 12 included studies reported statistically significant improvements in balance outcomes following intervention, irrespective of the type of training. However, a consistent trend was observed: multisensory balance training demonstrated superior effectiveness compared to conventional balance exercises in the majority of studies (see Table 1).

Summary of Evidence

Based on the findings summarized in Table 1, exercise-based interventions significantly improve balance in older adults. Multisensory training approaches appear to be more effective than conventional exercise programs, particularly in enhancing sensory integration, postural control, and neuromuscular coordination.

Discussion

The findings of this systematic review demonstrate that multisensory balance training yields superior outcomes to conventional exercise programs for improving balance, coordination, and functional mobility among older adults. While both approaches were effective in enhancing balance performance, multisensory interventions consistently showed greater improvements across standardized outcome measures, including the BBS, TUG, and FRT. These findings are consistent with the existing literature, which suggests that interventions targeting multiple sensory systems simultaneously lead to better functional outcomes and reduced fall risk in the geriatric population [4].

One possible explanation for the observed clinical improvements is that multisensory training may enhance the central nervous system's ability to integrate and prioritize sensory inputs from the visual, vestibular, and proprioceptive systems. With aging, these systems become less efficient, leading to impaired postural stability and delayed motor responses. Multisensory interventions help compensate for these deficits by enhancing sensory reweighting and promoting adaptive neural mechanisms [3].

Multisensory training enhances the central nervous system's ability to integrate and prioritize sensory inputs from visual, vestibular, and proprioceptive systems. This process, known as sensory reweighting, is essential for maintaining balance under varying environmental conditions. Training on unstable surfaces or with altered visual input forces the body to rely on alternative sensory pathways, thereby improving adaptability and stability [2,3]. These interventions improve neuromuscular coordination by promoting synchronized activation of muscle groups required for postural control. Enhanced coordination leads to more efficient motor responses, improved joint stability, and better control of the center of mass. Studies have shown that task-specific and multisensory exercises significantly improve motor planning and execution in older adults [6].

Multisensory training also enhances reactive postural control, which is critical for preventing falls during unexpected disturbances. Perturbation-based exercises expose individuals to controlled balance disturbances, improving their ability to generate rapid and appropriate corrective responses. This results in better recovery strategies and reduced fall risk [5].

Examples of multisensory training mechanisms: Foam surface training (proprioceptive challenge): Training on unstable surfaces, such as foam, reduces the reliability of somatosensory input from the feet and ankles, forcing the body to rely on vestibular and visual cues. This enhances proprioceptive sensitivity and improves postural stability under challenging conditions [2].

Visual cue training (motor planning): Visual feedback and cue-based exercises improve motor planning and anticipatory control by enhancing the individual's ability to interpret environmental information and adjust movements accordingly. This is particularly beneficial in tasks requiring dynamic balance and navigation [6].

Perturbation-based training (reactive balance): Perturbation training involves controlled external disturbances that challenge balance. This type of training improves automatic postural responses and reflexive stability mechanisms, enabling individuals to respond effectively to sudden loss of balance and reducing the likelihood of falls [5].

Clinical Interpretation

The consistent superiority of multisensory training observed in this review highlights the importance of task-specific, sensory-integrated rehabilitation approaches in geriatric physiotherapy. Unlike conventional exercises that primarily focus on strength and static balance, multisensory interventions mimic real-life challenges, thereby improving functional performance and independence (see Table 1).

Limitations

A considerable proportion of the included studies demonstrated "some concerns" or high risk of bias, particularly related to lack of blinding, inadequate allocation concealment, and absence of protocol registration. In rehabilitation-based research, blinding of participants and therapists is often challenging; however, the lack of assessor blinding may introduce measurement bias. These methodological limitations may affect the internal validity of the findings and should be considered when generalizing the results. Many of the included studies had relatively small sample sizes, which may limit the statistical power and increase the risk of type II error. Small samples also reduce the representativeness of the geriatric population, thereby limiting the external validity and generalizability of the findings to broader clinical settings. There was significant heterogeneity in the types, intensities, durations, and frequencies of interventions across studies. Multisensory training protocols varied widely, including differences in the use of unstable surfaces, visual feedback, and perturbation techniques. Similarly, conventional exercise programs were not standardized. This variability makes it difficult to determine the optimal intervention protocol and limits direct comparison across studies. Most studies focused on short-term outcomes, with limited or no follow-up assessments to evaluate the sustainability of improvements. As balance training aims to reduce fall risk and improve long-term functional independence, the absence of extended follow-up data restricts understanding of the durability of treatment effects. Reaction time was not consistently evaluated across the included studies, limiting the ability of this review to comprehensively assess the effect of multisensory exercises on this outcome. Therefore, the conclusions are mainly based on balance and functional mobility measures.

Future Recommendations

Future studies should focus on conducting well-designed RCTs with rigorous methodology, including proper randomization, allocation concealment, and blinded outcome assessment. This will enhance the reliability and validity of findings and strengthen the evidence base. There is a need to develop and implement standardized, replicable multisensory training protocols. Establishing consistency in intervention components, duration, and intensity will improve comparability across studies and help identify the most effective treatment strategies.

Future research should incorporate long-term follow-up assessments to evaluate the sustainability of treatment effects on balance, fall risk, and functional independence. This is crucial for determining the real-world clinical impact of multisensory interventions. Studies with larger sample sizes and diverse populations (e.g., community-dwelling vs. institutionalized elderly) are recommended to improve the generalizability and applicability of findings.

## Conclusions

The findings of this systematic review indicate that multisensory balance training is more effective than conventional exercise programs in improving balance, coordination, and functional mobility in the geriatric population. By targeting multiple sensory systems and enhancing neuromuscular coordination, multisensory interventions offer a more comprehensive approach to balance rehabilitation.

The integration of multisensory training into routine physiotherapy practice has the potential to significantly enhance postural control and improve overall functional independence among older adults. These interventions are particularly valuable in addressing the complex, multifactorial nature of balance impairment associated with aging. However, despite promising results, the current evidence is limited by methodological variability and lack of long-term data. Therefore, further high-quality research is essential to establish standardized protocols and confirm the long-term effectiveness of these interventions.
